# Cell-Type-Specific Adaptions in Striatal Medium-Sized Spiny Neurons and Their Roles in Behavioral Responses to Drugs of Abuse

**DOI:** 10.3389/fnsyn.2021.799274

**Published:** 2021-12-14

**Authors:** Marie-Charlotte Allichon, Vanesa Ortiz, Paula Pousinha, Andry Andrianarivelo, Anna Petitbon, Nicolas Heck, Pierre Trifilieff, Jacques Barik, Peter Vanhoutte

**Affiliations:** ^1^CNRS, UMR 8246, Neuroscience Paris Seine, Paris, France; ^2^INSERM, UMR-S 1130, Neuroscience Paris Seine, Institute of Biology Paris Seine, Paris, France; ^3^Sorbonne Université, UPMC Université Paris 06, UM CR18, Neuroscience Paris Seine, Paris, France; ^4^Université Côte d’Azur, Nice, France; ^5^Institut de Pharmacologie Moléculaire et Cellulaire, CNRS UMR 7275, Valbonne, France; ^6^Université Bordeaux, INRAE, Bordeaux INP, NutriNeuro, Bordeaux, France

**Keywords:** striatum, addiction, psychiatric disorders, synaptic plasticity, signaling, receptor tracking

## Abstract

Drug addiction is defined as a compulsive pattern of drug-seeking- and taking- behavior, with recurrent episodes of abstinence and relapse, and a loss of control despite negative consequences. Addictive drugs promote reinforcement by increasing dopamine in the mesocorticolimbic system, which alters excitatory glutamate transmission within the reward circuitry, thereby hijacking reward processing. Within the reward circuitry, the striatum is a key target structure of drugs of abuse since it is at the crossroad of converging glutamate inputs from limbic, thalamic and cortical regions, encoding components of drug-associated stimuli and environment, and dopamine that mediates reward prediction error and incentive values. These signals are integrated by medium-sized spiny neurons (MSN), which receive glutamate and dopamine axons converging onto their dendritic spines. MSN primarily form two mostly distinct populations based on the expression of either DA-D1 (D1R) or DA-D2 (D2R) receptors. While a classical view is that the two MSN populations act in parallel, playing antagonistic functional roles, the picture seems much more complex. Herein, we review recent studies, based on the use of cell-type-specific manipulations, demonstrating that dopamine differentially modulates dendritic spine density and synapse formation, as well as glutamate transmission, at specific inputs projecting onto D1R-MSN and D2R-MSN to shape persistent pathological behavioral in response to drugs of abuse. We also discuss the identification of distinct molecular events underlying the detrimental interplay between dopamine and glutamate signaling in D1R-MSN and D2R-MSN and highlight the relevance of such cell-type-specific molecular studies for the development of innovative strategies with potential therapeutic value for addiction. Because drug addiction is highly prevalent in patients with other psychiatric disorders when compared to the general population, we last discuss the hypothesis that shared cellular and molecular adaptations within common circuits could explain the co-occurrence of addiction and depression. We will therefore conclude this review by examining how the nucleus accumbens (NAc) could constitute a key interface between addiction and depression.

## Introduction

Drug addiction is defined as a compulsive pattern of drug-seeking/taking behavior despite damaging consequences, as well as a high rate of relapse after an abstinence period. Over the past years, research on preclinical models of addiction established that pathological behavior induced by addictive substances originate from long-lasting alterations of synaptic transmission (Lüscher and Janak, 2021). Drug-evoked changes in synaptic efficacy occur at specific synapses within discrete neuronal populations of the reward circuit and impact both the trafficking and functions of multiple neurotransmitter receptors, as well as downstream signaling pathways and gene expression programs (Nestler and Lüscher, 2019; Salery et al., 2020). This cell-type- and synapse-specific impact of drugs of abuse on synaptic and nuclear events shapes an enduring remodeling of the reward circuitry, which has been proposed to contribute to the transition from casual drug intake to addiction (Nestler, 2014; Lüscher and Janak, 2021).

A hallmark of drugs of abuse is to artificially increase dopamine concentration in the mesocorticolimbic system (Di Chiara and Imperato, 1988), which persistently modulates excitatory glutamate transmission within the reward circuit (Lüscher and Malenka, 2011), thereby hijacking natural reward processing (Keiflin and Janak, 2015). Studies on preclinical models of addiction, as well as Positron Emission Tomography (PET) imaging in humans, strongly support that behavioral alterations resulting from protracted drug exposure involve dopamine-evoked neuronal plasticity mechanisms taking place within the striatum, especially its ventral part, the nucleus accumbens (NAc) (Volkow and Morales, 2015; Salery et al., 2020). The NAc is indeed considered as a key target structure of addictive substances because it receives a dense glutamatergic innervation originating from limbic, thalamic and cortical regions, which encodes components of drug-associated stimuli and environment, along with dopamine projections from the ventral tegmental area (VTA) that mediates reward prediction error and incentive values (Hyman and Malenka, 2001).

The integration of converging glutamate and dopamine inputs by the striatum is critical for its central role in action selection execution, cognition, as well as reward-dependent learning and memory (Hyman and Malenka, 2001; Wickens et al., 2007). Signal integration is mostly achieved by the foremost neuronal population of the striatum that is composed of GABAergic medium-sized spiny neurons (MSN) receiving glutamate axon terminals and dopamine afferents converging onto their dendritic spines (Moss and Bolam, 2008; Doig et al., 2010). MSN primarily form two segregated populations based on the expression of either DA-D1 (D1R) or DA-D2 (D2R) receptors, which are G protein-coupled receptors positively and negatively coupled to adenylyl cyclase though their respective coupling to G_s/olf_ and G_i/o_ subtypes (Felder et al., 1991; Corvol et al., 2001). These two MSN subtypes display distinct projections within the cortico-basal ganglia network with the D1R-MSN and D2R-MSN forming the direct and the indirect pathway, respectively. The classical view is that the activation of direct pathway MSN promotes movement initiation, motivation and reinforcement learning, whereas the activation of the indirect pathway leads to opposite outcomes. Upon dopamine increase, the stimulation of D1R facilitates the activation of D1R-MSN, which promotes reinforcement whereas the stimulation of D2R inhibits D2R-MSN, thereby dampening their “anti-reward” functions (Gerfen and Surmeier, 2011).

Based on this model, it has been proposed that the surge of dopamine evoked in the NAc by drugs of abuse sets a high rewarding value of the drug and drug-associated cues as a consequence of concomitant stimulation of D1R and D2R, resulting in an imbalance between the activities of direct and indirect pathway neurons (Lobo and Nestler, 2011). However, recent evidence challenges a dichotomic model stipulating that the direct and indirect pathways play strictly opposite roles in motivated behavior, reward processing and addiction. In fact, about 5–15% of NAc MSN express both dopamine receptor (DAR) subtypes depending on NAc subregions (Bertran-Gonzalez et al., 2008) and roughly half of NAc D1R-MSN has been shown to project in the ventral pallidum (VP), which is the canonical output structure of indirect pathway MSN (Kupchik et al., 2015; Baimel et al., 2019; Pardo-Garcia et al., 2019). Furthermore, reciprocal lateral inhibitions between the two MSN subtypes has been described, locally in the striatum, and argue in favor of a synergistic, rather than dichotomic, role of direct and direct pathway MSN in motivated behavior (Burke et al., 2017). Accordingly, it has been shown that NAc D1R-MSN and D2R-MSN are concomitantly activated when rats are trained to press a lever to obtain a food reward (Natsubori et al., 2017). These few examples highlight the need to precisely characterize drug of abuse-evoked adaptations taking place in the two MSN populations and define their roles in controlling behavioral adaptations.

Current cell-type-specific approaches provided new insights into the cellular, molecular and morphological changes in identified neuronal populations, notably owing to the development of reporter mouse lines expressing fluorescent proteins or Cre-recombinase under the control of cell-specific promoters, *drd1a* and *drd2/Adora2a* for D1R-MSN and D2R-MSN, respectively (Gong et al., 2003, 2007; Lemberger et al., 2007; Shuen et al., 2008; Durieux et al., 2009; Valjent et al., 2009), combined with cell-type-specific manipulation of neuronal activity. Since most of the work done on cell-type-specific impacts of drugs is based on the use of psychostimulant, this review will be focused on studies using this class of addictive substances. We will review how the differential impact of drugs on MSN subtypes at the level of synaptic plasticity, receptor trafficking, protein–protein interactions and intracellular signaling, shapes persistent pathological behavior to psychostimulants. As a perspective, we will also discuss studies showing that multiple drug-evoked adaptations in NAc MSN strikingly parallel the ones observed in preclinical models of other psychiatric disorders, which could sustain the high comorbidity between addictions and other psychiatric disorders.

## Input-Specific and Drug-Evoked Synaptic Plasticity Changes Onto MSN Subpopulations

The transition from recreational to compulsive drug use has been proposed to involve a transition from ventromedial to dorsolateral striatal sub-regions (Belin et al., 2009; Everitt and Robbins, 2016). Therefore, long-lasting cellular and molecular alterations in the NAc and dorsomedial striatum (DMS) are believed to constitute a main step toward the development of addiction and most studies aiming at identifying the neuronal mechanisms of addiction have focused on these brain sub-regions. As detailed elsewhere (Salery et al., 2020), even if some subtleties exist depending on the type of neuronal manipulation (ablation, chemogenetic, and optogenetic manipulations), the tasks used (sensitization, conditioned-place preference, and self-administration) and the behavioral phases studied (acute responses, sensitization, withdrawal, extinction, and reinstatement), studies using cell-specific approaches to selectively manipulate the activity of MSN subpopulations broadly support the classical model of basal ganglia (Albin et al., 1989; DeLong, 1990; Kravitz et al., 2010), with D1R-MSN and D2R-MSN from the NAc and DMS playing antagonistic roles in psychostimulant-induced behavioral alterations. Briefly, ablation (Durieux et al., 2012), reversible blockade of synaptic transmission (Hikida et al., 2010), optogenetic inhibition (Ferguson et al., 2011; Chandra et al., 2013; Calipari et al., 2016) or chemogenetic silencing (Ferguson et al., 2011; Yager et al., 2019) of D1R-MSN tends to dampen behavioral adaptations to psychostimulants, while their optogenetic (Lobo et al., 2010) or chemogenetic (Heinsbroek et al., 2017) activation enhances these behavioral responses. In the case of D2R-MSN, their ablation (Durieux et al., 2009) or chemogenetic inhibition (Ferguson et al., 2011; Bock et al., 2013; Heinsbroek et al., 2017) tends to enhance psychostimulant-evoked adaptations, while their activation by chemogenetic (Farrell et al., 2013) or optogenetic (Lobo et al., 2010; Bock et al., 2013; Song et al., 2014) has opposite effects.

Few studies started to explore the output targets of MSN subpopulations involved in such psychostimulants-induced adaptations. For instance, selective inhibition of D1R-MSN-to-VP projections is sufficient to reduce reinstatement of cocaine seeking (Pardo-Garcia et al., 2019), while stimulation of putative D1R-MSN-to-lateral hypothalamus projections enhances drug seeking (Larson et al., 2015). In the same vein, depotentiation of D1R-MSN-to-VP projections after repeated cocaine exposure abolishes locomotor sensitization (Creed et al., 2016).

The aforementioned findings support the dichotomic view of D1R-MSN and D2-MSN playing antagonistic roles in drug-induced adaptations. Intriguingly, this goes against recent findings suggesting that the two MSN subtypes rather seem to act in a concerted fashion to control reward-related behavioral responses (Cui et al., 2013; Tecuapetla et al., 2016; Vicente et al., 2016; Sheng et al., 2019). In fact, optogenetic-mediated self-stimulation was described for both D1R-MSN and D2R-MSN of the NAc (Cole et al., 2018) and dorsolateral striatum (DLS) (Vicente et al., 2016). Comparably, inhibition of either MSN subpopulations in the NAc similarly increases unproductive reward seeking (Lafferty et al., 2020). In similar vein, transient optogenetic manipulations of both D1R-MSN and D2R-MSN in the NAc support a pro-motivational role for both cell types (Soares-Cunha et al., 2016, 2018; Natsubori et al., 2017; Tsutsui-Kimura et al., 2017). However, chemogenetic inhibition of NAc or DMS D2R-MSN rather potentiate performance in a motivational task (Carvalho Poyraz et al., 2016; Gallo et al., 2018). Such discrepancies between chemogenetic and optogenetic may stem from differences in duration and/or timing of D2R-MSN manipulations, but also from differential effects on feedback modulation by DA transmission (Olivetti et al., 2020; Soares-Cunha et al., 2020). Of note, activity and recruitment of MSN subpopulations in discrete phases of reward-seeking also seem to differ between sub-regions of the NAc (Tsutsui-Kimura et al., 2017). Altogether, these data regarding non-drug reward suggest a complex interplay between both MSN subpopulations for the control of reward processing rather than purely antagonistic actions, and call for caution regarding the interpretation of the results obtained through manipulations of MSN activity on drug-related behaviors since they could reflect perturbations of reward processing *per se*, rather than specific alterations of drug-induced behavioral adaptations. They also raise the intriguing idea that psychostimulant exposure may induce enduring alterations that bias MSN microcircuits toward a more dichotomic dynamic. This is in accordance with the observation that repeated cocaine exposure strengthens afferences from the basolateral amygdala onto D1R-MSN, but not D2R-MSN (MacAskill et al., 2014) and progressively enhances and dampens the activity of D1R-MSN and D2-MSN, respectively (van Zessen et al., 2021).

In this context, the work from the Luscher’s group was pioneer in establishing causal links between drug-induced synaptic adaptations onto D1R-MSN and specific behavioral alterations. They first showed that cocaine-induced locomotor sensitization is associated with a potentiation of cortical excitatory inputs onto NAc D1R-MSN, but not D2R-MSN, which optogenetic-mediated depotentiation reverses cocaine-induced sensitization (Pascoli et al., 2012). It has been proposed that the decrease in G protein-gated inwardly rectifying K+ (GIRK)-dependent signaling in the prelimbic cortex induced by repeated cocaine exposure (Hearing et al., 2013) may participate to cocaine-evoked increase of glutamate transmission onto D1R-MSN. Genetic ablation of GIRK channel activity in cortical pyramidal neurons has indeed been shown to selectively enhance AMPA receptor-dependent glutamate transmission onto NAc D1R-MSN and increase the motor effects of cocaine (Marron Fernandez de Velasco et al., 2017). Using cocaine self-administration, the Lüscher’s group further demonstrated that drug withdrawal induces a potentiation of cortical and hippocampal projections onto NAc D1R-MSNs. Strikingly, optogenetic manipulations of such projections during the withdrawal period was sufficient to reverse specific facets of cocaine self-stimulation, i.e., vigor and seeking when hippocampal and cortical inputs were manipulated, respectively (Pascoli et al., 2014b). More recently, they also established that compulsive self-stimulation of dopaminergic transmission – as a model of drug addiction – relies on the potentiation of excitatory projections from the orbitofrontal cortex onto D1R-MSNs of the dorsal striatum (Pascoli et al., 2018). This elegant set of studies highlights that discrete pathway-specific synaptic alterations mediate specific components of psychostimulant addiction and place D1R-MSN as the main subpopulation involved in such processes. However, increases in glutamate transmission onto D2R-MSN have also been reported after long access to a high dose of cocaine in the self-administration (Terrier et al., 2016) and in mice that have been trained for cocaine-administration but refrained from cocaine seeking when placed in an extinguished context (Roberts-Wolfe et al., 2018). However, further studies need to be conducted to unravel the contribution of changes in glutamate transmission onto D2R-MSN in drug-induced behavioral adaptations. This is a key issue since lateral inhibition of D2R-MSN onto D1R-MSN in the NAc has been suggested to play a main role in cocaine-induced locomotor sensitization (Dobbs et al., 2016; Burke et al., 2017), and recent work showed that cocaine-induced CPP is accompanied by a strengthening of the coupling between hippocampal place cells and D2R-MSN (Sjulson et al., 2018). Nonetheless, it is now timely to further explore molecular mechanisms underlying drug-evoked changes of synaptic efficacy at specific inputs projecting onto MSN subpopulations because it may contribute to the identification of relevant molecular targets for the treatment of addiction (Südhof, 2017).

## Cell-Type-Specific Signaling Triggered by Drugs of Abuse and Their Role in Behavioral Adaptations

The above described studies demonstrate that dopamine-mediated synaptic plasticity at specific glutamate input onto MSN shapes distinct components of behavioral adaptations to psychostimulants. Therefore, identifying the molecular basis underlying the imbalance between dopamine and glutamate transmission triggered by drugs appears as a major challenge, which may contribute to the development of innovative therapeutic strategies.

Because D1R and D2R are respectively coupled to G_s/olf_ and G_i/o_ subtypes, the increase of dopamine concentration induced by drugs in the NAc activates the cyclic adenosine monophosphate (cAMP) and downstream protein kinase A (PKA) pathway in D1R-MSN, while repressing it in D2R-MSN. When dopamine concentration rises, multiple PKA targets, including glutamate receptors, are therefore regulated in opposite ways in both MSN populations, which leads to a facilitation of glutamate-dependent activation of D1R-MSN and to its inhibition in D2R-MSN (Gerfen and Surmeier, 2011; Gardoni and Bellone, 2015; van Huijstee and Mansvelder, 2015). Accordingly, *in vivo* deep brain live calcium (Ca^2+^) imaging performed in the dorsal striatum of anesthetized *drd1a*-eGFP or *drd2*-eGFP mice showed that an acute cocaine injection produces a fast increase of intracellular Ca^2+^ in D1R-MSN, while a progressive decrease is observed in D2R-MSN (Luo et al., 2011). The modulation of both MSN’s activity by cocaine was shown to rely on the stimulation of DAR, therefore reflecting a DAR-dependent opposite modulation of neuronal activity of both MSN subtypes by cocaine. This was further confirmed by fiber photometry Ca^2+^ imaging in the NAc of freely moving mice where a single injection increases and decreases the frequency of Ca^2+^ transients in D1R-MSN and D2R-MSN, respectively. Of note, mice that have been conditioned in a cocaine-induced CCP paradigm displayed, on the test day, a transient Ca^2+^ rise in NAc D1R-MSN before entering the drug-paired compartment, while Ca^2+^ decreases in D2R-MSN when the animal stays in this compartment (Calipari et al., 2016). Altogether, these studies show that both cocaine and cocaine-associated cues modulate neuronal activity, and therefore Ca^2+^ signaling, in D1R-MSN and D2R-MSN in an opposite manner. This raised question as to the molecular mechanisms by which the stimulation of DAR controls glutamate-dependent excitation of MSN and Ca^2+^ signals in MSN in response to drugs.

*N*-methyl-D-aspartate receptors (NMDAR) are Ca^2+^ permeable ionotropic glutamate receptors that have long been recognized as essential for synaptic plasticity, as well as learning and memory (Paoletti et al., 2013). They are expressed by all MSNs and D1R stimulation has been shown to potentiate NMDAR functions (Flores-Hernández et al., 2002; Wittmann et al., 2005). The cell-type-specific role of NMDAR in psychostimulant adaptive behavior was first assessed by Heusner and Palmiter (2005) who generated a mouse line expressing in D1R-expressing cells a mutated version of the obligatory GluN1 subunit of NMDAR with reduced Ca^2+^ permeability. This manipulation prevented cocaine-induced locomotor sensitization and CPP (Heusner and Palmiter, 2005), therefore showing that NMDAR expressed in D1R-expressing neuron contribute to the development of the sensitizing and rewarding effects of cocaine. By contrast, the selective deletion of GluN1 in adenosine A2 receptor (A2AR)-expressing MSN (mostly overlapping with D2R-MSN) preserved amphetamine-mediated CPP (Lambot et al., 2016).

In support of an essential role of NAc NMDAR in drug-adaptive behavior, it has been shown that the inhibition of amphetamine-induced CPP and locomotor sensitization observed in mice bearing a deletion of GluN1 in all D1R-expressing neuron could be rescued by viral-mediated restoration of GluN1 expression in NAc D1R-MSN, or GluN1 deletion from all MSN (Beutler et al., 2011). Regarding the rewarding effects of cocaine, the deletion of GluN1 in D1R-, but not in A2AR-expressing cells, does not impact the development and extinction of cocaine-induced CPP but blunts cocaine-induced CPP reinstatement (Joffe et al., 2017). As observed for the sensitizing effects of cocaine (Beutler et al., 2011), the deletion of GluN1 in both D1R- and A2AR-expressing cells rescues the inhibition of CPP reinstatement of mice bearing a selective knock-out of GluN1 in D1R-expressing cells (Joffe et al., 2017). A balanced NMDAR signaling in D1R- and D2R-expressing cells seems therefore permissive for drug-adaptive behavior.

Altogether these studies support a functional role of the crosstalk between DAR- and NMDAR-dependent signaling in the NAc in long-lasting responses to psychostimulants. To dissect the molecular mechanisms underlying this crosstalk, we developed an *in vitro* model recapitulating main features of signaling events triggered *in vivo* in the striatum in response to acute cocaine exposure. Using cultured striatal neurons stimulated with a D1R agonist or a low dose of glutamate used separately or in combination (Pascoli et al., 2011; Cahill et al., 2014a), we showed that the stimulation of D1R triggers a cAMP-independent potentiation of NMDAR containing the GluN2B subunit. Upon D1R stimulation, the β/γ subunit of G_s/olf_ activates the tyrosine kinase Fyn, which in turn phosphorylates Tyr^1472^-GluN2B subunits of NMDAR (Pascoli et al., 2011). This phosphorylation event favors surface expression of NMDAR at synaptic sites (Hallett, 2006) and facilitates NMDAR-dependent Ca^2+^ signals (Pascoli et al., 2011). This crosstalk between D1R and NMDAR has a functional role since the inhibition of Fyn, or pharmacological blockade of GluN2B, blunts the development of cocaine-induced locomotor sensitization and CPP (Pascoli et al., 2011). Downstream from the receptors, the D1R-mediated potentiation of GluN2B-containing NMDAR triggers a Ca^2+^-dependent activation of the extracellular signal-regulated (ERK) pathways in the striatum in response to acute cocaine exposure (Pascoli et al., 2011), therefore explaining why cocaine-evoked ERK activation in the striatum requires the coincident stimulation of D1R and NMDAR (Valjent et al., 2000; Pascoli et al., 2011, 2014a; Cahill et al., 2014a,b). The ERK pathway is activated selectively in D1R-MSN (Bertran-Gonzalez et al., 2008) by virtually all drugs of abuse (Valjent et al., 2004) and its pharmacological blockade alters the long-term potentiation at cortical inputs onto D1R-MSN (Pascoli et al., 2012). ERK activation in the striatum also controls morphological changes of D1R-MSN induced by cocaine (Ren et al., 2010; Kim et al., 2011; Dos Santos et al., 2017), as well as the development of the sensitizing and rewarding effects of this drug (Valjent et al., 2000, 2006b) and the reconsolidation of drug-paired contextual cues memories (Miller and Marshall, 2005; Valjent et al., 2006a). The key role of the ERK pathway in persistent drug-adaptive behavior relies on the fact that, once activated in dendrites, ERK translocates to the nucleus (Trifilieff et al., 2009), where it launches epigenetic and transcriptional regulations in D1R-MSN that have been exhaustively reviewed elsewhere (Cahill et al., 2014b; Pascoli et al., 2014a; Salery et al., 2020). Downstream from D1R and NMDAR, the activity of the Wiskott-Aldrich syndrome protein family verprolin homologous protein 1 (WAVE1), which controls actin polymerization, is also modulated by cocaine exposure. Cell-type-specific manipulation of WAVE1 expression revealed that the selective role of WAVE1 signaling in D1R-, but not in D2R-expressing neurons, in both the development of cocaine-induced CPP and the maintenance of locomotor sensitization, as well as in cocaine-evoked feedback regulation of glutamate transmission onto D1R-MSN (Ceglia et al., 2017).

The canonical cAMP/PKA pathway recruited downstream from D1R stimulation also participates to the potentiation of NMDAR through the direct targeting of NMDAR subunits (Flores-Hernández et al., 2002; Cepeda and Levine, 2006). This pathway also indirectly modulates the amplitude of striatal ERK activation and shapes behavioral responses to psychostimulants in a cell-type-specific manner through the targeting of the cAMP-regulated phosphoprotein (DARPP-32). Once phosphorylated by PKA on Thr^34^, activated DARPP32 inhibits the protein phosphatase 1 (PP1), which reinforces the phosphorylation of MEK and ERK (Valjent et al., 2005). Cell-type-specific analysis of DARPP32 activity revealed that a PKA-dependent activation of DARPP32 occurs in D1R-MSN in response to cocaine, whereas its cyclin-dependent kinase 5 (cdk-5)-mediated inhibition is detected in D2R-MSN (Bateup et al., 2008), which is consistent with the selective activation of ERK in D1R-MSN. At the behavioral level, deletion of DARPP32 in D1R-MSN or D2R-MSN respectively blunts and increases responses to cocaine (Bateup et al., 2010). The fact that DARPP32 constitutive inactivation in all MSN (Valjent et al., 2005) reduces responses to psychostimulants, suggests that activation of cAMP/PKA/DARPP32 pathways in D1R-MSN predominates over DARPP32 signaling in D2R-MSN to drive drug-adaptive behavior. Activation of the cAMP/PKA/DARPP32 also leads to a PP1-mediated inhibition of the striatal-enriched tyrosine phosphatase (STEP) targeting ERK, therefore reinforcing ERK activation downstream from D1R. STEP is also involved in the crosstalk between D1R- and NMDAR-signaling since STEP promotes NMDAR endocytosis, through the dephosphorylation of GluN2B subunits and therefore modulates the duration of NMDAR-mediated ERK activation (Paul et al., 2003). However, the cell-type-specific function of STEP in D1R-MSN and D2R-MSN in drugs of abuse-induced behavioral adaptations has not been addressed.

The integration of dopamine and glutamate receptor-dependent signaling therefore controls a complex intracellular signaling network, more precisely characterized in D1R-MSN than in D2R-MSN, which drives electrophysiological, morphological and behavioral long-term effects of psychostimulants. As a consequence, the targeting of dopamine and glutamate receptors has been envisioned to alleviate addiction symptoms in human. However, this approach is associated with a loss of efficacy over time and the appearance of deleterious side effects (Cahill et al., 2014b), likely due to the implication of dopamine and glutamate receptors in multiple physiological functions. This observation supports that there is need for the identification of new strategies to inhibit the dialogue between dopamine and NMDA receptors.

## Roles of Dopamine Receptor Heteromerization in Drugs of Abuse-Evoked Behavioral Responses

As discussed above, preclinical models of drug exposure established that distinct phases of drug-adaptive behaviors are driven by dopamine-evoked modulation of synaptic transmission at specific glutamate inputs onto MSN subpopulations (Pascoli et al., 2012, 2014b,^2018^; MacAskill et al., 2014; Creed et al., 2016). This calls for a better understanding of the precise molecular events underlying the detrimental interplay between dopamine and glutamate signaling triggered by drugs of abuse. In this context, growing evidence supports that receptor heteromerization, which corresponds to direct physical interaction between receptors of distinct subtypes, is a potent mechanism by which receptors can reciprocally fine-tune their functions. The rising interest for these receptor heteromers mostly stems from their functional properties that are distinct from the individual component receptors. Their ability to dynamically modulate binding affinity of component receptors and downstream signaling pathways positions receptor heteromers as targets to consider for the development of more selective pharmacological treatment for multiple neurological and psychiatric disorders (Fiorentini et al., 2003; Cristina et al., 2006; Wang et al., 2012; Borroto-Escuela et al., 2017). Dopamine receptors have been shown to form oligomers with multiple partner receptors (Andrianarivelo et al., 2019). This chapter will be focused on studies that have provided evidence for a functional role of endogenous heteromers formed between dopamine and glutamate receptors in the striatum on drug-adaptive behavior.

Heteromers formed between DAR and NMDAR have been the subject of intense investigations and are considered as key players for the integration of converging dopamine and glutamate signals (Andrianarivelo et al., 2019). Seminal studies from Fang Liu’s group established the existence of direct physical interactions between C-terminal ends of D1R and GluN1 subunits of NMDAR in rat hippocampal tissues (Lee et al., 2002). These heteromers were later detected in the striatum *in vivo* (Cahill et al., 2014a), including at synapses (Fiorentini et al., 2003). The stimulation of NMDAR has been shown to increase the presence of D1R in dendritic spines of MSN through a mechanism involving D1R-GluN1 interaction (Scott et al., 2006), therefore suggesting that D1R-NMDAR complexes are formed within, or at close proximity, of synapses. Functionally, D1R-NMDAR heteromerization is required for the potentiation of NMDA-mediated post-synaptic currents by a D1R agonist, as well as for electrically evoked long-term potentiation of cortical inputs onto D1R-MSN (Cahill et al., 2014a). These latter results therefore position D1R-NMDAR heteromers as molecular bridges linking changes in dopamine transmission to the modulation of glutamate synapses impinging onto D1R-MSN. By contrast, the direct binding of D2R to GluN2B subunits of NMDAR has been shown to mediate the inhibition of NMDAR by dopamine in D2R-MSN (Liu et al., 2006). These observations raise questions about the potential involvement of those two heteromers subtypes in the activation of D1R-MSN and inhibition of D2R-MSN triggered by drug-evoked increase in dopamine. To address this issue, the impact of repeated exposures to psychostimulant on DAR-NMDAR interactions has been studies in both MSN subtypes. We observed that cocaine-induced locomotor sensitization is associated with an increased heteromerization of both D1R-NMDAR and D2R-NMDAR in the NAc (Andrianarivelo et al., 2021). However, the dynamics of these receptor interactions differs since cocaine-evoked D1R-NMDAR heteromerization appears as a transient phenomenon, whereas D2R-NMDAR heteromerization in the NAc core subdivision outlasts a one-week abstinence period. Owing to a temporally controlled disruption of D1R-NMDAR heteromerization in the NAc, it was shown that D1R-NMDAR interactions are required for cocaine-induced ERK activation and for the potentiation of glutamate transmission onto D1R-MSN induced by repeated cocaine exposures. At the behavioral level, inhibiting D1R-NMDAR selectively blocks the development of cocaine-induced locomotor sensitization and CPP but preserves the maintenance of these behaviors. By contrast, inhibiting D2R-NMDAR complexes alters the maintenance of cocaine’s sensitizing effects as well as the reinstatement of cocaine-induced CPP (Andrianarivelo et al., 2021), therefore showing that DAR-NMDAR heteromerization in both MSN subtypes shapes distinct components of psychostimulant-adaptive behaviors. Notably, the implication of these heteromers depends on the nature of the reward since disrupting either heteromer subtype in the NAc preserves food reward processing (Andrianarivelo et al., 2021). The involvement of D2R-NMDAR heteromerization in controlling the maintenance of the sensitizing and rewarding effects of cocaine without impacting on natural reward processing points those heteromers as targets of potential therapeutic value. This assumption is reinforced by the fact that such heteromers are also detected in human post-mortem caudate putamen samples from subjects with psychostimulant use disorder and matched controls (Andrianarivelo et al., 2021). Notably, we found a sharp decrease of D2R protein contents in samples from individuals with psychostimulant use disorder, which could partly explain the diminished bioavailability of D2R described in psychostimulant-dependent subjects by PET-imaging (Volkow et al., 1999; Koob and Volkow, 2016; Trifilieff et al., 2017). However, despite this decreased D2R expression, the remaining pool of D2R forming D2R-NMDAR heteromers was threefold higher in subjects with psychostimulant misuse when compared to matched controls (Andrianarivelo et al., 2021), therefore reinforcing that targeting D2R-NMDAR heteromers may be a strategy to pursue in order alleviate addiction symptoms in human.

In addition to NMDAR, D1R has been shown to form heteromers with D3R both *in vitro* in heterologous systems and *in vivo* in the NAc (Fiorentini et al., 2008; Marcellino et al., 2008), which holds potential interests in preclinical models of addiction. The D3R is enriched in the shell subdivision of the NAc and is predominantly expressed in D1R-MSN (Sokoloff et al., 1990; Schwartz et al., 1998). Pharmacological activation of D3R potentiates the rewarding effects of cocaine (Caine and Koob, 1995), whereas D3R blockade has the opposite effects (Xi et al., 2005). Accordingly, mice bearing a deletion of D3R display an enhanced acute locomotor response to cocaine, an increased sensitivity to the rewarding effects of amphetamine (Xu et al., 1997), as well as an enhancement of both cocaine-self administration and motivation for cocaine-taking/seeking behavior (Song et al., 2012), therefore supporting that alterations of D3R signaling represent a vulnerability factor toward psychostimulant addiction. D1R-D3R interaction has been shown to increase upon stimulation of both receptors with dopamine and to reciprocally modulate component receptor functions (Fiorentini et al., 2008). Allosteric interactions within this heterodimer are associated with a positive cross-talk between both receptors that drives a downstream beta-arrestin 1-biased signaling triggering ERK activation and promoting locomotor responses (Marcellino et al., 2008; Guitart et al., 2014, 2019). Even though D3R expression and D1R-D3R heteromers have been shown to increase in preclinical models of L-DOPA induced dyskinesia and to promote dyskinesia (Farré et al., 2015; Solis et al., 2017), further work is needed to characterize the potential implication of D1R-D3R heteromers in drug-evoked long-term adaptations.

## Drug-Evoked Morphological Changes in Medium-Sized Spiny Neurons Subtypes and Regulation of Synaptogenesis

Psychostimulant-evoked connectivity changes of dopaminoceptive neurons may contribute to the long-lasting effects of psychostimulants. In fact, acute and repeated cocaine injections, as well as cocaine self-administration, trigger an increase of MSN dendritic spine density (Golden and Russo, 2012; Marie et al., 2012; Dos Santos et al., 2017), which has been correlated to an increase in synapse density, as detected by electron microscopy after repeated cocaine exposure (Alcantara et al., 2011). The enhancement of spine density is cell-type-specific as it is confined to D1R-MSN (Dobi et al., 2011; Kim et al., 2011). Multiple strategies, including knock-out, pharmacological dissections, and virally mediated knock-down have been used to identify the molecular bases of this structural plasticity (Russo et al., 2010; Golden and Russo, 2012). The picture that has emerged is the one of pluralism, with multiple molecular pathways appearing as essential for cocaine-induced increased spine density. Extracellular signals, including BDNF or glutamate and dopamine acting in synergy, activate signaling pathways such as Akt (Cahill et al., 2016) or ERK (Ren et al., 2010), which impact on neuronal morphology by modulating actin dynamics through monomeric G proteins such as Rac1 (Dietz et al., 2012). By targeting transcription factors, these same signaling pathways also regulate transcriptional levels of others kinases, such as CDK5 (Kumar et al., 2005) or CaMKII (Robison et al., 2013), which are themselves controlling structural plasticity (Norrholm et al., 2003). Redundancy between components of this complex signaling network is exemplified by the observation that selective inhibition of the transcription factors Elk1 (Besnard et al., 2011) or CREB (Brown et al., 2011), which are activated downstream from the ERK pathway, fully blocks cocaine-evoked increase in MSN dendritic spine density. Extracellular matrix proteins and cell–cell adhesion also play an essential role in cocaine-induced increase in spine density, notably through integrins (Chen et al., 2008; Wiggins et al., 2009; Kerrisk and Koleske, 2013), SynCAM1 (Giza et al., 2013), or thrombospondin (Wang et al., 2021).

Those studies have been conducted on fixed cerebral tissues, although spine formation involves distinct steps with spine growth, eventually followed by spine maintenance. A time-lapse study allowed to distinguish between these two steps and unraveled distinct underlying molecular mechanisms that are both dependent ERK activation. Spine formation was followed in D1R-MSN by two-photon microscopy in adult striatal slices after co-stimulation with glutamate and a D1R agonist, a co-stimulation paradigm recapitulating the main molecular events triggered by cocaine *in vivo* (Pascoli et al., 2011). This approach established that ERK activation, but neither transcription nor translation, was essential for spine growth. By contrast, the ERK-mediated activation of the cytoplasmic kinase MNK-1, as well as translation, but not transcription, was required for spine maintenance during the hour following spine growth. Hence, spine growth relies on ERK acting on cytoplasmic targets, while early spine maintenance could rely on an ERK-mediated activation of local translation since MNK-1 is known to target the translation initiation factor eiF4E (Bramham et al., 2016). However, long-term spine maintenance involves transcriptional regulation since the blockade of ERK-dependent activation of the transcription factor Elk-1 inhibits spine density increase (Besnard et al., 2011). These findings therefore support a three-step model by which the rapid activation of ERK induced by cocaine initially instructs spine growth, likely through the targeting of cytoplasmic substrates. In a second step, ERK activates local translation through MNK-1 for early spine stabilization and ERK activation finally drives gene expression, notably *via* the targeting of Elk-1, which is required for long-term spine maintenance.

An open question is whether the increased spine density observed after repeated injections is solely due to the first injection, or whether each injection would induce new spines. A single cocaine administration leads to a rapid and long-lasting increase in spine density, which is detectable as early as 1-h post-cocaine exposure and lasts as long as 1 month after cocaine administration (Dos Santos et al., 2017). The fact that the increase in MSN spine density is not higher after chronic regimen compared to single injection may indicate that the enhancement of spine density after chronic cocaine is not the simple sum of repeated waves of spine growth. Accordingly, *in vivo* time-lapse imaging in the prefrontal cortex revealed that each cocaine injection induces spine formation, with some spines being eliminated and others being maintained, drawing a progressive increase in density reaching an overall stable increased density after five cocaine injections (Muñoz-Cuevas et al., 2013). Along those lines, a meta-analysis of articles in which spine density was measured after various regimens of cocaine administration (Anderson and Self, 2017) reported that, while all studies observed increased spine density after repeated cocaine injections, the only studies reporting long-lasting increase in spine density used protracted regimens of cocaine exposure. This observation supports that, while a single cocaine injection induces spine density increase, not all spines are stabilized and repeated injections induce repeated spine growth phases leading to more persistent increase in spine density. Although comparing the percentages of dendritic spine increase between studies is difficult, it also appears that the amplitude of this increase is not lower after a single injection as compared to chronic regimens, therefore suggesting that the repetition of cocaine administration does not scale up spine density but would allow the persistence over time of newly formed spines. Structural plasticity thus seems a progressive process through repetition of cocaine administration leading to a stabilized increased spine number on D1R-MSN dendrites.

Another important question is how psychostimulant-evoked changes in spine density relate to functional plasticity? An important parameter to consider for this question is also the size of the spine, more specifically the volume of the spine head, since it correlates with synaptic transmission efficacy (Holler et al., 2021). In the case of a single cocaine administration, the increased spine head volume (Dos Santos et al., 2017) correlates with increased synaptic events in activated c-Fos-positive MSN (Koya et al., 2012) and long-term potentiation (Pascoli et al., 2012). However, the picture is far less clear for other regimens of cocaine administration. Chronic cocaine injection induces a reduction of AMPA/NMDA currents ratio (Kourrich et al., 2007) as a consequence of decreased AMPA currents at single synapse (Khibnik et al., 2016), which seems in contradiction with an increased spine density. Following withdrawal from cocaine, the AMPA/NMDA currents ratio is then increased (Kourrich et al., 2007), while spine density remains stable. One possible explanation for this discrepancy is that chronic cocaine induces silent synapses, which have been shown to become active after withdrawal. Hence, while a first cocaine injection would create more dendritic spines and potentiate glutamatergic transmission, repeated injection would induce new spines hosting silent synapses that would become active upon deprivation (Boudreau et al., 2007; Graziane et al., 2016). Along those lines, some studies reported that chronic cocaine increases the percentage of spines with small size, which is compatible with an increase of AMPAR-lacking silent synapses (LaPlant et al., 2010; Dietz et al., 2012; Cahill et al., 2016; Wang et al., 2021), whereas an enhancement of dendritic spine heads has been reporter after a protracted withdrawal from cocaine (Graziane et al., 2016). By contrast, other studies reported either no changes (Dumitriu et al., 2012; Heck et al., 2015) or an increase (Dobi et al., 2011) in spine size 1 or 2 days after the last cocaine injection. Nevertheless, a prevailing hypothesis is that an increase in density of small spines followed by an increase in density of larger spines may correspond to cocaine-evoked initial generation of silent synapses followed by their unsilencing during prolonged withdrawal (Wright et al., 2020).

The above-described studies focus on the impact of psychostimulant on dendritic spine density, size and shape. A key question is how such changes in dendritic spines relate to drug-induced alterations in striatal connectivity? Spines are protrusions hosting glutamatergic post-synapse, an increased spine density implies a change in synaptic integration without bringing information on the origin of the afferents. Both D1R-MSN and D2R-MSN share similar morphology and have the same spine density in the dorsal striatum (Suárez et al., 2014; Suarez et al., 2016; Gagnon et al., 2017) as well as in the NAc (MacAskill et al., 2014). The first dendritic segment is devoid of spines, usually ending with a branching point. Spine density is then constant throughout the dendrite, only the very distal part showing a higher density. Glutamate afferents impinging onto MSN’s spines in the dorsal striatum originate from neocortex, thalamus, with very few afferents from the amygdala; while in the NAc glutamate inputs come from neocortex, thalamus, basolateral amygdala, and ventral hippocampus (Sesack and Grace, 2010). Projection neurons from the thalamus express the vesicular Glutamate Transporter of type 2 (VGLUT2), while the other afferents express VGLUT1. In the dorsal striatum, the proportion of spines connected with neocortical VGLUT1 boutons or with thalamic VGLUT2 boutons is similar and this applies to glutamate inputs onto both D1R-MSN and D2-MSN (Doig et al., 2010). However, while all VGLUT1 boutons contact spines, 70% of VGLUT2 boutons contact spines and 30% of VGLUT2 boutons make synapse on the dendritic shaft (Doig et al., 2010). Analysis of dendrites in a knock-in mouse allowing the visualization of all VGLUT1 boutons revealed that 50% of spines are in contact with VGLUT1 boutons in both the core (Heck et al., 2015) and the shell (Dos Santos et al., 2017) subdivisions. Furthermore, spines in contact with VGLUT1 and VGLUT2 boutons are uniformly distributed along the dendrite (Dos Santos et al., 2017). This was confirmed by viral labeling of afferents from prefrontal cortex, amygdala, and thalamus, which has revealed that those afferents do not contact spines in a clustered manner (Xia et al., 2020). Upon acute and chronic cocaine administration, the enhancement of spine density is associated with an increase in spines contacting VGLUT1- and VGLUT2-expressing pre-synaptic boutons in the NAc (Heck et al., 2015; Dos Santos et al., 2017). Interestingly, the percentage of spines in contact with VGLUT1 boutons remains constant, meaning that the ratio of spines in contact with VGLUT1 and VGLUT2 afferents is not modified upon exposure to cocaine. Since afferents from prefrontal cortex, amygdala, and ventral hippocampus all express VGLUT1, further studies are needed to depict the respective contribution of those structures to connectivity change on to MSN subpopulations. However, the viral-mediated labeling of amydalar afferents, coupled to manipulations of their activity through optogenetic, revealed that inhibition of those afferents blunted cocaine-evoked spine density increase, which, however, does not exclude a similar necessity for other afferents (MacAskill et al., 2014).

Dopaminergic afferents also undergo structural plasticity upon cocaine administration. The majority of dopaminergic boutons do not contact MSN dendrites (Descarries et al., 1996), but their density is such that they are never located further than 1micron from a glutamate synapse (Moss and Bolam, 2008). However, 20% of MSN spines have a dopaminergic bouton apposed to their neck (Moss and Bolam, 2008; Dos Santos et al., 2018). Cocaine-induced locomotor sensitization has been shown to correlate with an increase in dopaminergic boutons in the NAc shell subdivision, with a strong increase in the percentage of spines displaying dopaminergic boutons apposed to the neck of MSN dendritic spines (Dos Santos et al., 2018). This remodeling of dopaminergic connectivity may have an important role in modulating plasticity at glutamate synapse since the release of DA within a short-time window has been shown to unlock the head spine size increase induced by glutamate (Yagishita et al., 2014). One long-standing question remains as whether spines bearing dopaminergic boutons apposed to their neck have distinct integrative properties, and whether those spines are preferentially connected with specific glutamate afferents. Recent studies highlighted that glutamate synapses onto MSN originating from distinct brain regions display distinctive properties (Deroche et al., 2020) and are differentially modulated by dopamine (Christoffel et al., 2021). However, further work is needed to determine if these functional differences recorded by electrophysiology have a morphological counterpart.

The mode of formation of striatal synapses may also have relevant implications for rules of connectivity. While an increased density in MSN dendritic spine contacting VGLUT1 boutons and dopaminergic boutons has been reported in response to repeated cocaine administration, no change in the density of VGLUT1 boutons was detected. This implies that newly formed spines contact already existing boutons, which had been proposed as a possible mode of synaptogenesis in the adult brain (Holtmaat and Svoboda, 2009). Glutamatergic boutons making synapses with two spines has been reported in the striatum by electron microscopy (Doig et al., 2010), and the occurrence of two spines in contact with a single bouton is increased after cocaine exposure (Heck et al., 2015; Dos Santos et al., 2017). In addition, time-lapse analysis of synaptogenesis in adult striatum slice showed that new spines contact VGLUT1 boutons present before initiation of spine growth (Dos Santos et al., 2017). The formation of synapses on preexisting boutons could have important implications for the integrative properties of the striatal network. The case of two spines from the same dendrite connected to a common presynapse implies temporal summation of synaptic inputs arising from the same glutamatergic neuron. In the case of a common presynapse for two dendritic spines from two distinct MSN, the newly formed spine would imply an increase in the number of neurons making inputs to a MSN, rather than an increase in the number of inputs arising from the same glutamatergic neuron. Indeed, in the striatum, boutons along the glutamatergic axon are separated by 10 micrometers on average and exhibit rather linear geometry (Kincaid et al., 1998; Zheng and Wilson, 2002), it is therefore unlikely that a glutamatergic axon builds several connections with one MSN dendrite. Dendritic spines have been widely studied in the context of drugs of abuse, bringing insights in their morphological changes, rules of formation and the associated molecular mechanisms. It should however be noted that a putative causal link between dendritic spine changes and drug-induced behavior remains an open question as long as the methods by which spine changes are blocked interfere with other cellular functions.

## Stress-Induced Adaptations and Their Commonalities With Drug-Induced Remodeling: Toward the Identification of Shared Molecular Substrates

This review provided so far compelling evidence for cell-type-specific adaptations in striatal MSN, their role in the neurobiological impact of psychostimulants on the brain, and how they contribute to substance use disorder (SUD). Although SUD are complex multifactorial psychiatric conditions, stressful experiences constitute a primary factor that can potentiate responses to most licit and illicit abused substances and therefore favor the appearance of addictive behaviors (Sinha, 2001). Importantly, stress has been widely acknowledged to increase drug-craving states, which are a deterrent to quit drug use, and risks of relapse when one individual is experiencing negative life experiences or when exposed to stress-associated cues (George and Koob, 2017; Wemm and Sinha, 2019).

Epidemiological and clinical data indicate that people suffering from SUD often experience, at least, a second psychiatric conditions, distinct from drug abuse, such as mood disorders. When two pathologies occur, simultaneously or sequentially, in the same person, they are described as comorbid. In addition to time correlation, comorbidity also implies interactions between the illnesses that affect the course and prognosis of both. A major finding in psychopathology research is that comorbidity is the rule rather than the exception. Comorbid SUD and psychiatric disorders are indeed commonly encountered in our modern society. However, they often lack adequate treatment and are mostly associated with poor prognosis. Therefore, comorbidity has emerged as a major clinical, public, and research issue (Dani and Harris, 2005). The high prevalence of co-occurrence of two psychiatric conditions is likely to reflect common, or at least partially overlapping, neuronal substrates as well as genetic and environmental factors. As for SUD, the likelihood of developing mood disorders is also largely influenced by one’s ability to cope with stressful challenges (Riboni and Belzung, 2017). Hence, stress stands as a core component for virtually all psychiatric conditions and therefore a close examination of the impact of stress on the brain is essential to understand the potential underpinning mechanisms contributing to SUD and comorbid psychiatric diseases. The hypothalamo-pituitary adrenal (HPA) axis is the pivot for the stress response. Over activation of the HPA axis can rapidly switch processes from adaptive to maladaptive, and hence, the stress response can favor numbers of mental conditions and impede their remission; for example, exposure to trauma and stress increases the risks of depressive episodes and facilitates their recurrence. Interaction with modulatory systems is key to apprehend how stress impairs coping with the external world. The noradrenergic system has been historically the primary focus of stress network. However, a core component of mental disorders implicates alterations of reward processes that are now consistently associated with stress reaction (Marinelli and McCutcheon, 2014; Vaessen et al., 2015; Pignatelli et al., 2017). Indeed, stress-induced alterations of the reward circuitry have been reported in both humans and rodents (Russo and Nestler, 2013). In rodents, a causal link has been established between the dysregulation of VTA DA neurons activity and the appearance of depressive-like symptoms including social withdrawal and anhedonia (Barik et al., 2013; Chaudhury et al., 2013). Increased release of dopamine with the NAc has been observed following acute social defeat, repeated forced swim test or exposure to threatening social cues following chronic social defeat (CSD; Tidey and Miczek, 1996; Lemos et al., 2012; Barik et al., 2013). Notably, this modulation of dopamine release relied on the release of corticotropin releasing hormones, and its action at both CRF type 1 and 2 receptors, as well as glucocorticoids, the ultimate actor of the HPA axis, *via* activation of glucocorticoid receptors.

Several models of stress with various degrees of duration and intensity have been developed in order to mimic symptoms reminiscent of human mood disorders including depression (Gray et al., 2015). Although these models cannot fully replicate the complexity of the human condition, they allow to study key aspects of the pathology and to understand the underlying mechanisms. Amongst these models, the CSD model, which induces depressive-like symptoms in mice susceptible to stress, allows the possibility of studying resiliency to stress as a subgroup of mice submitted to CSD do not develop any sign of the pathology (Golden et al., 2011).

As largely evidenced in the sections related to drugs of abuse, dopamine has a key role in filtering excitatory inputs to the NAc. Similarly, stress has been shown to dysregulate excitatory inputs to the NAc in a projection-specific manner. Indeed, an enhancement of thalamic-NAc and ventral hippocampal-NAc synaptic transmission was observed in mice susceptible to CSD (Bagot et al., 2015; Christoffel et al., 2015; Muir et al., 2020). Conversely, the stimulation of mPFC and amygdala inputs promoted resilience to stress (Bagot et al., 2015). When examining the impact of CSD on AMPA/NMDA ratio, increases were observed only at thalamic-NAc, but not PFC-NAc, synapses (Christoffel et al., 2015). Although, clearly revealing a complex impact of stress at a circuit level, these studies did not address whether these changes in synaptic transmission occurred in specific MSNs subpopulations.

Examining stress-induced cell-type-specific adaptations in the NAc, chronic restraint stress was shown to decrease AMPA/NMDA ratio in D1R-expressing neurons, while D2.R-expressing neurons remained unaffected (Lim et al., 2012). The authors have used electrical stimulation to measure AMPA/NMDA ratio therefore it remains to be determined if these adaptations occur at selective inputs onto the NAc. These changes in synaptic efficacy were driven by α-melanocyte-stimulating hormone *via* its action on the melanocortin type 4 receptor (Lim et al., 2012). Targeting this signaling pathway *via* Sh-RNA was sufficient to reverse stress-induced anhedonia (Lim et al., 2012). Within this line of research, CSD stress was shown to decreased excitatory synaptic inputs to D1R-MSN and increased in D2R-MSN in susceptible mice (Francis et al., 2015). Furthermore, this defeat protocol triggered changes in the intrinsic properties and *in vivo* activity of D1R-MSN; indeed, increased excitability of D1R-MSN was reported following CSD. Activation of D1R-MSN *via* optogenetic stimulation reversed social aversion, while their pharmacogenetic silencing conferred stress susceptibility (Francis et al., 2015). In contrast, manipulating D2R- MSN’s activity, either enhancing or decreasing, failed to alter behavioral responses to CSD (Francis et al., 2015). These differences in D1R-MSN vs D2R-MSN responsiveness were also reported using exposure to mild repeated foot shocks (Pignatelli et al., 2021). Increased AMPA/NMDA ratio were observed in D1R-MSNs and reflected an increase in AMPAR currents without alterations in NMDAR-mediated responses in stressed mice when compared to control group. These stress-induced potentiation at D1R-MSNs synapses occurred at inputs originating from the ventral hippocampus and were associated to the appearance of anhedonia and passive coping (Pignatelli et al., 2021). Strikingly, these changes in synaptic plasticity were absent in D2R-MSNs (Pignatelli et al., 2021). Regardless of the nature of the stressor, which varied from physical to psychological, D1R- and D2R-MSN are differently affected by stressful experiences. To date, D1R-MSN appear cardinal in the appearance of symptoms reminiscent of psychiatric conditions such as depression and traumatic memories.

Scarce studies examined the impact of stress on inhibitory synapses within the NAc. Recently, a decreased expression of neuroligin-2 was reported in post-mortem tissue analyses from the NAc of depressed patients (Heshmati et al., 2018). These findings were of importance since, in contrast to other members of the neuroligin family of cell adhesion molecules, neuroligin-2 expression is restricted to inhibitory synapses (Ali et al., 2020). Mirroring the clinical data, CSD triggered a down regulation of neuroligin-2 expression (Heshmati et al., 2018). This decrease was restricted to D1R-MSN as CSD failed to alter its level within D2R-MSN. Further evidence for an impact of stress on NAc inhibitory synapses arose from the study of Heshmati et al. (2020) who demonstrated a decrease in vesicular GABA transporter and gephyrin in postmortem NAc tissues from both depressed patients and socially defeated mice, without determining whether one MSN cell-type was preferentially affected (Heshmati et al., 2020). However, the expression of at least three key molecular components of inhibitory synapses were shown to be modulated by stress and therefore constitute a strong impetus to further investigate how stressful life experiences can alter the balance between excitation and inhibition with the NAc.

Alterations in MSN cellular architecture has been associated with dopamine-dependent neuronal adaptations (Alberquilla et al., 2020), especially in the context of drug administration (see previous section). This structural plasticity contributes to a stable rewiring of synaptic connections impinging on the flow of information within delineated brain networks. Using sholl analysis, susceptible mice were shown to present reduced dendritic length and branch points in D1R-, but not D2R-expressing neurons (Francis et al., 2017; Fox et al., 2020). Yet, the spine density remained unaffected in D1R-MSNs, while it appeared enhanced in D2R-MSNs (Francis et al., 2017; Fox et al., 2020). These changes were specific to the NAc as morphology of D1R-MSNs remained unaltered in the dorsal striatum (Fox et al., 2020). Importantly, when probing for both cell-type- and spine-type-specific comparisons, no changes in synaptic strength were reported in either D1R-MSN or D2R-MSN subpopulations of susceptible mice contrasting with the impact of cocaine (Khibnik et al., 2016). Changes in synaptic strength only occurred in resilient mice, which exhibit an increase of mushroom-shaped spines in D1R-MSN and a decrease in D2R-MSN. A better understanding of the mechanism underpinning these functional adaptations in resilient mice may help design new treatments to ease depressive symptoms.

The differential impact of stress on D1R-MSN and D2R-MSN are also found at the molecular and epigenetic levels. Indeed, alterations in gene expression programs underpin enduring physiological and behavioral adaptations that contribute to the appearance of depression symptomatology. Recently, a whole-genome sequencing of patients with recurrent major deficit disorders identified a significant increase of the signal for the *SIRT1* locus, which encodes a histone deacetylase (CONVERGE consortium, 2015). Increases in SIRT1 is observed following CSD highlighting the translational importance of the findings. Its selective deletion or over-expression in D1R-MSN produced increased and decreased stress coping, respectively, while modulating its expression in D2R-MSN had no consequence on stress outcome (Kim et al., 2016). Of interest, in light of the impact of cocaine on signaling cascades is the modulation by stressful stimuli of the PKA signaling cascade and recruitment of key proteins such as DFosB and CDK5. Along those lines, Plattner et al. (2015) found that the D1-MSN-specific knock-out of CDK5 engendered increased cAMP levels and PKA activity, as well as the appearance of resilience to chronic social stress and increased stress coping in response to acute stressors (Plattner et al., 2015). ΔFosB expression has long been known to be sensitive to chronic stress exposure (Hope et al., 1994), but it is only recently that a more detailed cell-specific analyses of its expression have been revealed. Increased expression of ΔFosB was observed in D1R-MSN of resilient mice (Lobo et al., 2013) and its overexpression in this MSN subtype promotes resiliency (Vialou et al., 2010). In contrast, in D2R-MSN, increased ΔFosB levels were observed in susceptible animals following CSD (Lobo et al., 2013). This balance between resilience and susceptibility relies, at least in part, on the epigenetic regulation of the *fosb* gene with distinct contribution of acetylation and methylation in D1R-MSN and D12R-MSN (Hamilton et al., 2018).

Overall, this review highlights the complexities of adaptations induced by natural and addictive rewards triggered within the NAc emphasizing the differential contribution of D1R-MSN and D2 MSNs. We described how stress also highjacks the reward system to induce adaptations at specific inputs to the NAc and also in a cell-type-specific manner. Although, much work is needed to build a comprehensive landscape of the enduring changes occurring following these stimuli, the development of viral tools in order to probe for cell- and projection-specific adaptations has already allowed to decipher some of the key sequences of events that underlie pathological states based on the deregulation of the reward system. Facing the high prevalence of comorbidities between SUD and other psychiatric disorders, with about 50% of patients suffering from one condition also developing the other, it has been proposed that brain dysfunctions underlying SUD and other mood disorders, such as depression, may share partly common pathophysiological mechanisms. Future work should therefore focus on the identification of common molecular events that are essential for SUD and other psychiatric conditions. This is in line with the recent push toward transdiagnostic approach of psychiatric disorders and recommendations, notably from the NIDA, stressing that therapeutic developments should focus on both SUD and comorbid mood disorders, rather than treating each disease independently, which holds poor efficacy (Ross and Peselow, 2012; Kelly and Daley, 2013).

## Author Contributions

All authors listed have made a substantial, direct, and intellectual contribution to the work, and approved it for publication.

## Conflict of Interest

The authors declare that the research was conducted in the absence of any commercial or financial relationships that could be construed as a potential conflict of interest.

## Publisher’s Note

All claims expressed in this article are solely those of the authors and do not necessarily represent those of their affiliated organizations, or those of the publisher, the editors and the reviewers. Any product that may be evaluated in this article, or claim that may be made by its manufacturer, is not guaranteed or endorsed by the publisher.
